# Spontaneous Browning of White Adipose Tissue Improves Angiogenesis and Reduces Macrophage Infiltration After Fat Grafting in Mice

**DOI:** 10.3389/fcell.2022.845158

**Published:** 2022-04-26

**Authors:** Jiayan Lin, Shaowei Zhu, Yunjun Liao, Zhuokai Liang, Yuping Quan, Yufei He, Junrong Cai, Feng Lu

**Affiliations:** Department of Plastic and Cosmetic Surgery, Nanfang Hospital, Southern Medical University, Guangzhou, China

**Keywords:** fat grafting, browning, beige adipocytes, angiogenesis, inflammation

## Abstract

**Background**: Fat grafting is a frequently used technique; however, its survival/ regeneration mechanism is not fully understood. The browning of white adipocytes, a process initiated in response to external stimuli, is the conversion of white to beige adipocytes. The physiologic significance of the browning of adipocytes following transplantation is unclear.

**Methods**: C57BL/6 mice received 150 mg grafts of inguinal adipose tissue, and then the transplanted fat was harvested and analyzed at different time points to assess the browning process. To verify the role of browning of adipocytes in fat grafting, the recipient mice were allocated to three groups, which were administered CL316243 or SR59230A to stimulate or suppress browning, respectively, or a control group after transplantation.

**Results**: Browning of the grafts was present in the center of each as early as 7 days post-transplantation. The number of beige cells peaked at day 14 and then decreased gradually until they were almost absent at day 90. The activation of browning resulted in superior angiogenesis, higher expression of the pro-angiogenic molecules vascular endothelial growth factor A (VEGF-A) and fibroblast growth factor 21 (FGF21), fewer macrophages, and ultimately better graft survival (Upregulation, 59.17% ± 6.64% vs. Control, 40.33% ± 4.03%, *p < 0.05), whereas the inhibition of browning led to poor angiogenesis, lower expression of VEGF-A, increased inflammatory macrophages, and poor transplant retention at week 10 (Downregulation, 20.67% ± 3.69% vs. Control, 40.33% ± 4.03%, *p < 0.05).

**Conclusion**: The browning of WAT following transplantation improves the survival of fat grafts by the promotion of angiogenesis and reducing macrophage.

## 1 Introduction

Autologous fat grafting is a procedure for soft-tissue reconstruction and repair (Khouri et al., 2014a; Pu et al., 2015). Although there are a growing number of techniques to optimize the outcome of fat grafting, the quality of the transplanted fat tissue remains unpredictable (Khouri and Khouri 2017). Therefore, the mechanisms by which fat grafts survive following transplantation are under investigation.

Early angiogenesis, immune cells, and activated adipose stem cells (ASCs) have been reported to have effects on the survival of transplanted fat tissue (Chappell et al., 2015; Hong et al., 2018; Cai et al., 2018a). Accumulating evidence suggests that inflammatory cells and ASCs both play important roles during fat grafting, but the behavior of mature adipocytes has been less well studied.

Wu et al. were the first to identify a distinct type of thermogenic adipocyte within subcutaneous white adipose tissue (WAT), which was called a beige adipocyte (Wu et al., 2012). The conversion of white to beige adipocytes in white fat depots is referred to as “browning process.” Subsequent studies have shown that WAT undergoes browning after transplantation and that the “browned” area is accompanied by a marked increase in angiogenesis (Qiu et al., 2018). This browning process occurred in human fat grafting as well but the significance of that is unknown (Liu et al., 2021). The administration of either tamoxifen or extracellular vesicles derived from ASCs to stimulate browning of WAT results in an improvement in fat graft survival and retention (Cai et al., 2018a; Zhu et al., 2020). However, both of these promote WAT browning indirectly, instead of by directly targeting the white adipocytes and the thermogenic gene program. Therefore, the direct relationship between the browning of white adipocytes and fat graft survival, and the underlying mechanisms, remain to be identified.

White adipocytes in WAT depots, and especially in subcutaneous depots, are able to be converted to beige adipocytes in response to specific stimuli (Rosenwald et al., 2013). Unlike classical brown adipose tissue (BAT), which expresses uncoupling protein-1 (UCP1) at a high level and has a high capacity for respiratory uncoupling, beige adipocytes demonstrate a lower level of thermogenic gene expression in the basal state (Petrovic et al., 2010). However, differences among adipose tissue types cannot only be attributed to their thermogenic program. In addition to differences in metabolism, the secretome of the various types of adipose tissue significantly varies (Villarroya et al., 2017a). Thermogenic adipose tissue secretes various factors that control the expansion of adipose tissue, whereas the secretome of WAT principally consists of molecules that are not normally released by BAT (Cannon and Nedergaard 2004; Ortega et al., 2011). BAT has been shown to produce vascular endothelial growth factor A (VEGF-A), which promotes local vascularization, particularly during cold exposure (Xue et al., 2009). Moreover, upon thermogenic stimulation, beige adipose tissue secretes fibroblast growth factor 21, a cytokine previously reported to normalize glucose and lipid homeostasis as well as promote angiogenesis (Yaqoob et al., 2014; Huang et al., 2017, Huang et al., 2019; Zhou et al., 2019; Dai et al., 2021). Furthermore, the transplantation of beige precursor cells is associated with the development of larger numbers of blood vessels in a mouse model of delayed repair of rotator cuff tear. This implies that beige adipocytes in transplanted fat might improve gr (Schulz et al., 2011)aft survival by secreting factors that activate angiogenesis (Lee et al., 2020).

Previous studies have shown that various treatments can be used to activate browning in fat tissue and that these significantly improve fat graft survival (Cai et al., 2018b; Zhu et al., 2020). However, the role of the spontaneous browning of WAT following transplantation is poorly understood, as is the mechanism by which beige adipocytes influence fat graft retention. To define the role of adipocyte browning in fat graft retention and study the underlying mechanism by which browning affects fat graft survival, we studied the effects of the β3 adrenoceptor agonist CL316243, which directly tragets the β3 adrenoceptor on white adipocytes and subsequently induces browning (Schulz et al., 2011; Montanari et al., 2017), and those of the β3 adrenoceptor antagonist SR59230A, which inhibits browning (Jiang et al., 2017), on the degree of browning at various time points in a mouse model of fat transplantation.

## 2 Materials and Methods

### 2.1 Animals and Treatments

The study was approved by the Nanfang Hospital Animal Ethics Committee and was conducted according to the guidelines of the National Health and Medical Research Council of China. Sixty-eight 8-week-old male C57BL/6 mice were obtained from Southern Medical University (Guangzhou, China). For transplantation, the mice were anesthetized by the intraperitoneal injection of pentobarbital sodium (50 mg/kg). One hundred fifty milligrams of fat tissue was harvested from the inguinal fat pad, cut into small pieces, and inserted through a 5 mm-long skin incision into a small dorsal subcutaneous pocket in 32 mice, which was followed by skin closure with 7-0 nylon sutures. After 7, 14, 30, and 90 days, *n* = 8 mice per time point were sacrificed and the transplanted adipose tissue was collected for further analyses. In a second experiment, to investigate the role of browning after transplantation, 36 mice were randomly allocated to three groups (*n* = 12 per group): a browning upregulation group, a browning downregulation group, and a control group. The browning upregulation group was administered CL316243 (APExBIO, Houston, TX, United States) (1 mg/kg body mass) subcutaneously at the site of transplantation for 14 consecutive days following transplantation, the browning downregulation group was administered SR59230A (MedChemExpress, NJ, USA; 1 mg/kg body mass) in the same way, and the control group was administered phosphate-buffered saline (PBS) (5 ml/kg body mass). After 2 or 10 weeks, the mice were killed and the fat grafts were harvested and analyzed.

### 2.2 Histology and Immunohistochemical Staining

Samples were fixed in 4% paraformaldehyde for 24 h, embedded in paraffin and cut from the middle. Sections were prepared and stained with hematoxylin and eosin, and examined under a light microscope (Olympus BX51, Tokyo, Japan). Paraffin-embedded tissue sections were incubated with rabbit anti-UCP1 antibody (dilution 1:2,000; Abcam, Cambridge, UK) or rabbit anti-cluster of differentiation (CD)31 (1:1,000; Abcam), followed by secondary antibody and then an avidin-biotin-horseradish peroxidase detection system (ZSGB-BIO, Beijing, China). The slides were then examined using an Olympus BX51 microscope. For immunofluorescence staining, tissue sections were incubated with rabbit anti-mouse macrophage antigen 2 (MAC2) (1:150; Abcam), or guinea pig anti-mouse perilipin (dilution 1:400; Progen Biotechnik, Heidelberg, Germany). For double fluorescence staining, sections were incubated with AlexaFluor 488-conjugated (Thermo Fisher Scientific, Waltham, MA, United States) goat anti-guinea pig immunoglobulin G (1:200; Abcam), and AlexaFluor rhodamine-conjugated chicken anti-rabbit immunoglobulin G (1:200; Invitrogen, Carlsbad, CA, United States). Nuclei were stained with 4′,6-diamidino-2-phenylindole (Sigma, St. Louis, MO, United States). To quantify the degree of browning, at least five microscopic fields per samples of grafts were randomly chosen from the middle to the edge by two of the authors in a double-blinded fashion. To quantify the proinflammatory cells, and angiogenesis in the transplanted fat tissue, the numbers of UCP-1-positive cells, Mac-2-positive cells, and CD31-positive cells were counted in at least five microscopic fields per sample by two of the authors in double-blinded fashion. The percentage of perilipin positive adipose cells area per field was determined with the use of ImageJ (National Institutes of Health, Bethesda, Md.). The diameter of mature adipocytes was determined by analyzing the captured image of mature adipocytes by means of light microscopy with the use of the ImageJ (National Institutes of Health, Bethesda, Md.) program as described previously (Wankhade et al., 2016).

### 2.3 Quantitative RT-PCR

Adipose tissue was collected, frozen in liquid nitrogen, and stored at −80°C. RNA was extracted from 50 mg tissue samples using RNeasy Lipid Tissue Mini Kits (Qiagen, Hilden, Germany), according to the manufacturer’s instructions. cDNA was synthesized and amplified over 40 cycles using a QuantiTect Reverse Transcription Kit (Qiagen) and a Rotor-Gene 3000 Real-Time PCR Detection System (Corbett Research, Sydney, Australia). Expression levels were calculated using the 2−ΔΔCt method. The primer sequences were as follows: Ucp1: forward 5′-CTG​ATG​AAG​TCC​AGA​CAG​ACA​G-3′ and reverse 5′-CCA​GCA​TAG​AAG​CCC​AAT​GA-3′; PRDM16: forward 5′-CAG​CAC​GGT​GAA​GCC​ATT​C-3′ and reverse 5′-GCG​TGC​ATC​CGC​TTG​TG-3′; PGC1-α: forward 5′-CGA​CAG​CTA​TGA​AGC​CTA​TGA​G-3′ and reverse 5′-CTT​CTG​CCT​CTC​TCT​CTG​TTT​G-3′; FGF21: forward: 5′-CTG​GGG​GTC​TAC​CAA​GCA​TA-3′ and reverse 5′-CAC​CCA​GGA​TTT​GAA​TGA​CC-3′; and VEGF-A: forward 5′-GGA​GAT​CCT​TCG​AGG​AGC​ACT​T-3′ and reverse 5′-GGCGATTTAGCAGCAGATATAAGAA-3′HIF1α: forward: 5′-CAA​GAT​CTC​GGC​GAA​GCA​A-3′ and reverse 5′-GGT​GAG​CCT​CAT​AAC​AGA​AGC​TTT-3′.

### 2.4 Energy Expenditure Analysis Using the Comprehensive Lab Animal Monitoring System

Oxygen consumption (VO_2_) and carbon dioxide expiration (VCO_2_) were collected and measured by indirect calorimetry in a Promethion Metabolic Screening System (Sable systems International, United States) that maintained constant environmental temperature across a 12-h light, 12-h dark cycle. The mice in different groups were individually housed in chambers maintained at 25°C, with free access to food and water. Measurements were made every 15 min for 3 days after the mice had acclimatized to their surroundings for 1 day. Mean values were calculated for all the parameters of interest over the 3 days of monitoring.

### 2.5 Statistical Analysis

Statistical analyses were performed using SPSS version 22.0 (IBM, Inc., Armonk, NY, United States). The data are expressed as mean ± SEM and were compared among the groups using one-way analysis of variance or the Kruskal-Wallis test. Comparisons between two groups were performed using the least significant difference method or the Mann-Whitney U-test. *P* < 0.05 was considered to represent statistical significance.

## 3 Results

### 3.1 Early Accumulation of Beige Adipocytes in the Center of the Grafts

**FIGURE 1:**
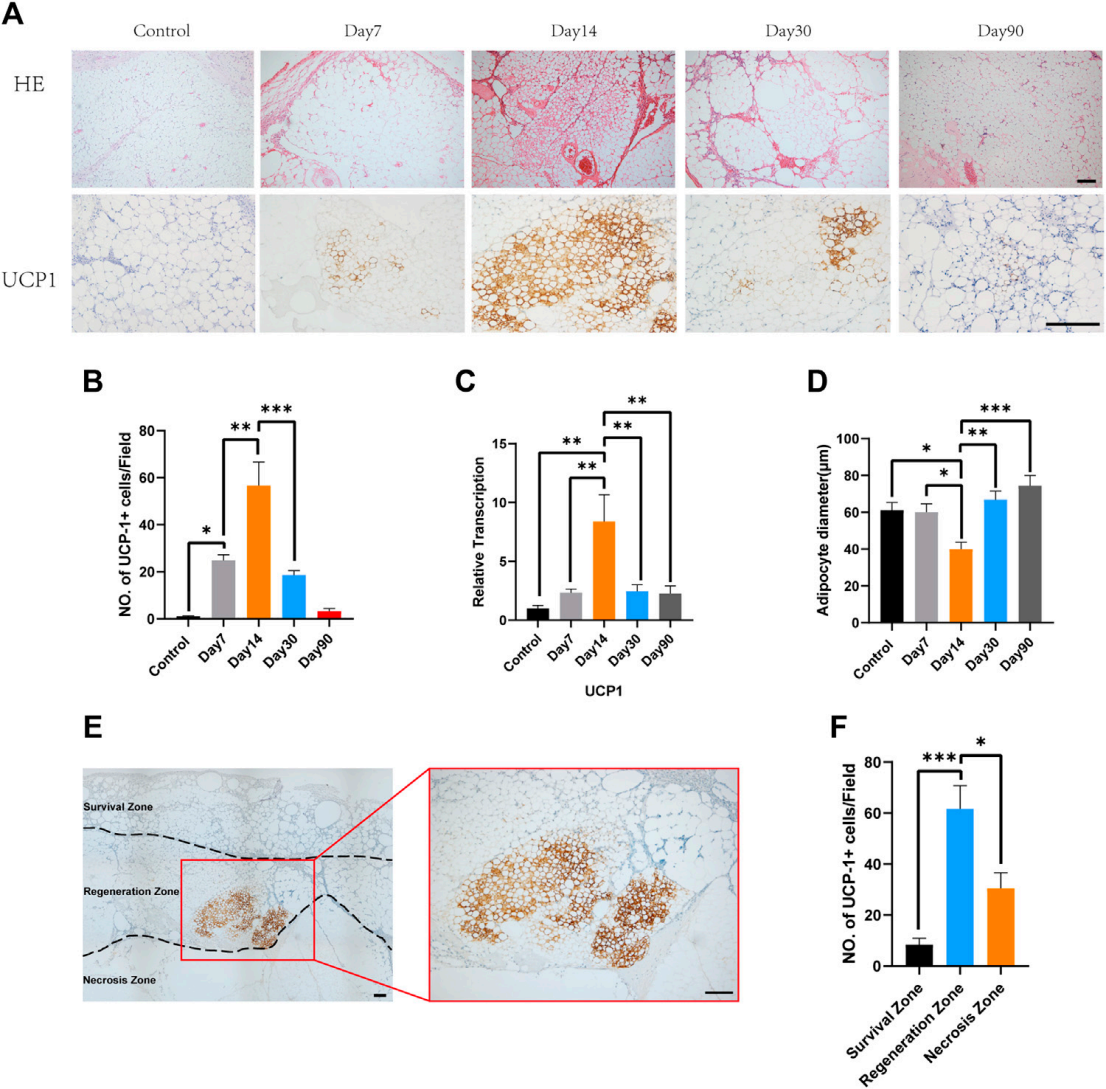
Time course of the accumulation of beige adipocytes in the center of fat grafts. **(A)** Fat grafts on day 14 contain smaller adipocytes and more multilocular adipocytes and show strong immunostaining for uncoupling protein (UCP)-1. On days 7 and 30, there were more unilocular adipocytes and fewer UCP-1-positive fat cells, and still fewer on day 90. Presented figures were from the same zone of each grafts. **(B)** Beige cell counts at the various time points. **(C)** Expression of Ucp1 on days 7, 14, and 30. **(D)** On day 14, mean adipocyte diameter was lower than on day 7, 30 and 90. **(E)** Representative photomicrograph of UCP1 immunostaining, showing beige adipocytes in the “regeneration zone” of a graft. **(F)** Beige cell count in survival zone, regeneration zone and necrosis zone. (*p < 0.05;**p < 0.01;***p< 0.001). Scale bar = 100 μm.

In the present study, to better characterize the timing of the appearance of beige adipocytes, we analyzed the fat grafts at various time points after transplantation. Hematoxylin and eosin staining showed that number of multilocular adipocytes peaked at day 14 while adipocytes in fat graft contained only one single lidpid droplets at day 7, 30 and 90 (Figure 1A, above). Immunohistochemistry showed that a large number of UCP-1-positive adipocytes had accumulated by day 14 and that fat grafts harvested on days 7, 30, and 90 mostly comprised large adipocytes with single lipid droplets and few UCP1-positive adipocytes (Figure 1A, below). The number of UCP1-positive cells peaked 14 days after surgery, with fewer beige adipocytes being present on days 7, 30, and 90 (Control, 1.0 ± 0.32 vs. Day 7, 24.8 ± 2.41 vs. Day 14, 56.6 ± 10.08 vs. Day 30, 18.6 ± 1.89 vs. Day 90, 3.2 ± 1.16, *p < 0.05; **p < 0.01; ***p < 0.001) (Figure 1B). Ucp1 gene expression, measured using quantitative reverse transcription polymerase chain reaction, was also highest in fat grafts harvested on day 14 (Control, 1.0 ± 0.26 vs. Day 7, 2.34 ± 0.31 vs. Day 14, 8.38 ± 2.28 vs. Day 30, 2.45 ± 0.56 vs. Day 90, 2.27 ± 0.63, **p < 0.01) (Figure 1C). Mean adipocyte size was significantly lower on days 14 compared with that at day 7, day 30 and day 90 (Control, 61.05 ± 4.31 vs. Day 7, 60.06 ± 4.58 vs. Day 14, 39.99 ± 3.76 vs. Day 30, 66.91 ± 4.65 vs. Day 90, 74.37 ± 5.61, *p < 0.05; **p < 0.01; ***p < 0.001) (Figure 1D). Based on the knowledge that depending on the distance from the surface, adipocyte fate after transplantation is categorized into three zones including survival zone, regeneration zone and necrosis zone, we define the zone where most of beige adipocytes accumulated. Immunohistochemistry for UCP1 showed that browning tended to occur in the regeneration zone of the fat grafts, rather than in the survival zone (Figure 1E). Counting of the UCP1+ adipocytes indicated that there were significantly more beige adipocytes in the regeneration zone than in the survival zone or necrosis zone (Survival zone, 8.4 ± 2.46 vs. Regeneration zone, 61.6 ± 9.1 vs. Necrosis zone, 30.4 ± 6.15, *p < 0.05;***p < 0.001) (Figure 1F).

### 3.2 Pharmacologic Downregulation or Upregulation of Browning Affects Graft Retention and Fat Graft Structure

**FIGURE 2:**
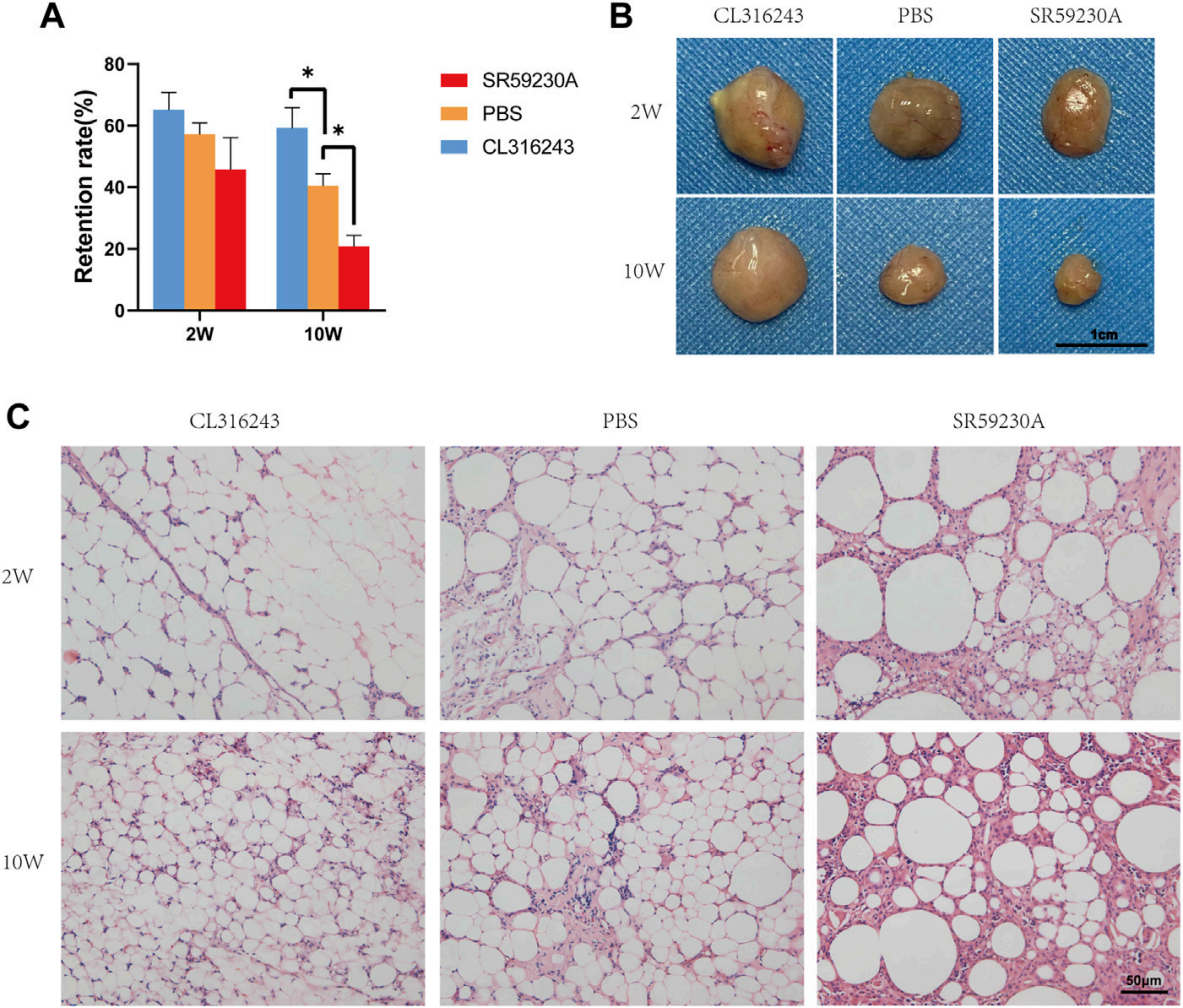
Retention (final mass/initial mass) and adipose structure of the transplanted fat in the three groups. **(A)** After 2 weeks, there was no significant difference, but after 10 weeks, retention was significantly better in the browning upregulation group (CL316243) than in the control group (PBS) and the browning downregulation group (SR59230A) showed significantly poorer retention than the control group. **(B)** Swelling of the transplanted adipose tissue was found in the upregulation group but not in the control group and downregulation group at week 2; however, the grafts in the downregulation and control groups obviously shrank at week 10. **(C)** Histologic analysis showed that fat grafts from the control group had normal adipose structure 10 weeks after transplantation, whereas samples from CL316243-treated mice had superior structure. However, grafts from the browning upregulation group demonstrated small, brown-like adipocyte features, whereas grafts from the browning downregulation group contained more oil cysts, larger adipocytes, and crown-like structures. (*p < 0.05)

Two weeks after transplantation, no significant difference was observed among the retention rate of fat grafts from three groups (Figure 2A). However, 10 weeks after transplantation, the fat graft volume retention of the browning upregulation group was superior to that of the control group (Upregulation, 59.17% ± 6.64% vs. Control, 40.33% ± 4.03%, *p < 0.05) (Figure 2A). And the browning downregulation group has worse retention rate than that of the control group (Downregulation, 20.67% ± 3.69% vs. Control, 40.33% ± 4.03%, *p < 0.05) (Figure 2A). Swelling of the transplanted adipose tissue was found in the upregulation group but not in the control group and downregulation group at week 2; however, the grafts in the downregulation and control groups obviously shrank at week 10 (Figure 2B). Hematoxylin and eosin staining showed that 2 weeks after transplantation, no significant difference between upregulation group and control group was observed while some of oil cysts existed in fat grafts in downregulation group (Figure 2C, above). 10 weeks following transplantation, there were larger numbers of oil cysts and more severe fibrosis in the downregulation group than in the control group (Figure 2C, below). By contrast, the samples from the browning upregulation group had superior adipose structure with less oil cysts or inflammatory cells (Figure 2C, below). .

### 3.3 Pharmacologic Treatment Modifies the Browning of the Fat Grafts

**FIGURE 3:**
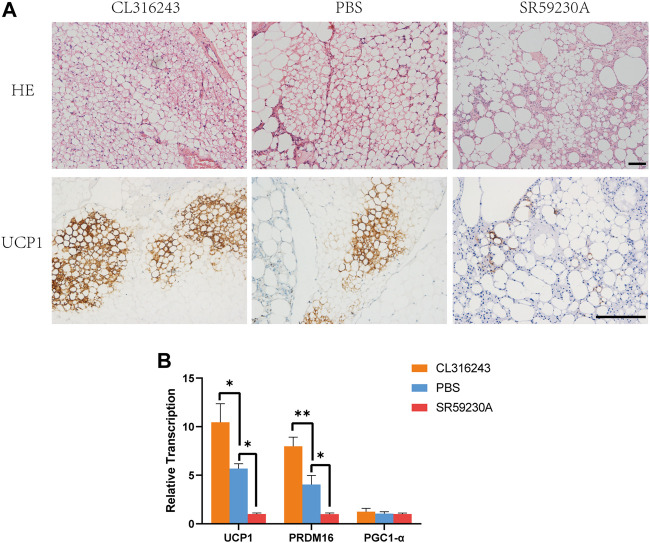
Manipulation of the degree of browning of the grafts using CL316243 and SR59230A. **(A)** Histologic analysis showed that fat grafts from the upregulation group contained smaller and more multilocular adipocytes than the control group and downregulation group 2 weeks after transplantation. (above) Immunohistochemistry for the beige adipocyte marker uncoupling protein (UCP)-1 showed a larger number of UCP1-positive beige adipocytes in the upregulation group and a smaller number in the downregulation group, compared with the control group. (below) **(B)** There was a similar trend for the expression of browning-related genes (Ucp1 and Prdm16). There were no significant differences in the expression of Pgc1a among the groups. (*p < 0.05;**p < 0.01). Scale bar = 50 μm.

The grafts were harvested from mice in the three groups after treatment with the browning stimulator or inhibitor for 14 consecutive days. Hematoxylin and eosin staining showed that CL316243 treated fat grafts containing multiple, small lipid droplets than the control group and downregulation group (Figure 3A, above). Immunohistochemistry showed that grafts from the browning upregulation group had larger numbers of UCP1-positive beige cells (Figure 3A, below). However, the induction of browning was significantly inhibited in fat grafts from SR59230A-treated mice vs. control mice (Figure 3A, below). There was significantly higher expression of Ucp1 and Prdm16 mRNA in the upregulation group than in the control group (Figure 3B) and significantly lower expression in the downregulation group than in the control group (Figure 3B). No significant difference of PGC1-α mRNA expression was observed among different groups (Figure 3B). Two weeks of treatment of both CL316243 and SR59230A had no influence on weight gain and food intake (Supplementary Figures S1A, B). Besides, oxygen consumption, CO_2_ production, and heat production were not affected by CL316243 and SR59230A (Supplementary Figures S1C, D and E). Local administration, but not intraperitoneal injection, of the agonist and the antagonist might explain why there was no significant difference among different groups. Also, lack of sufficient blood vessels in grafts at this early stage could not facilitate the delivery of substances throughout the body.

### 3.4 Browning is Associated with Higher Expression of VEGF-A and FGF21 and Superior Angiogenesis in the Fat Grafts

**FIGURE 4:**
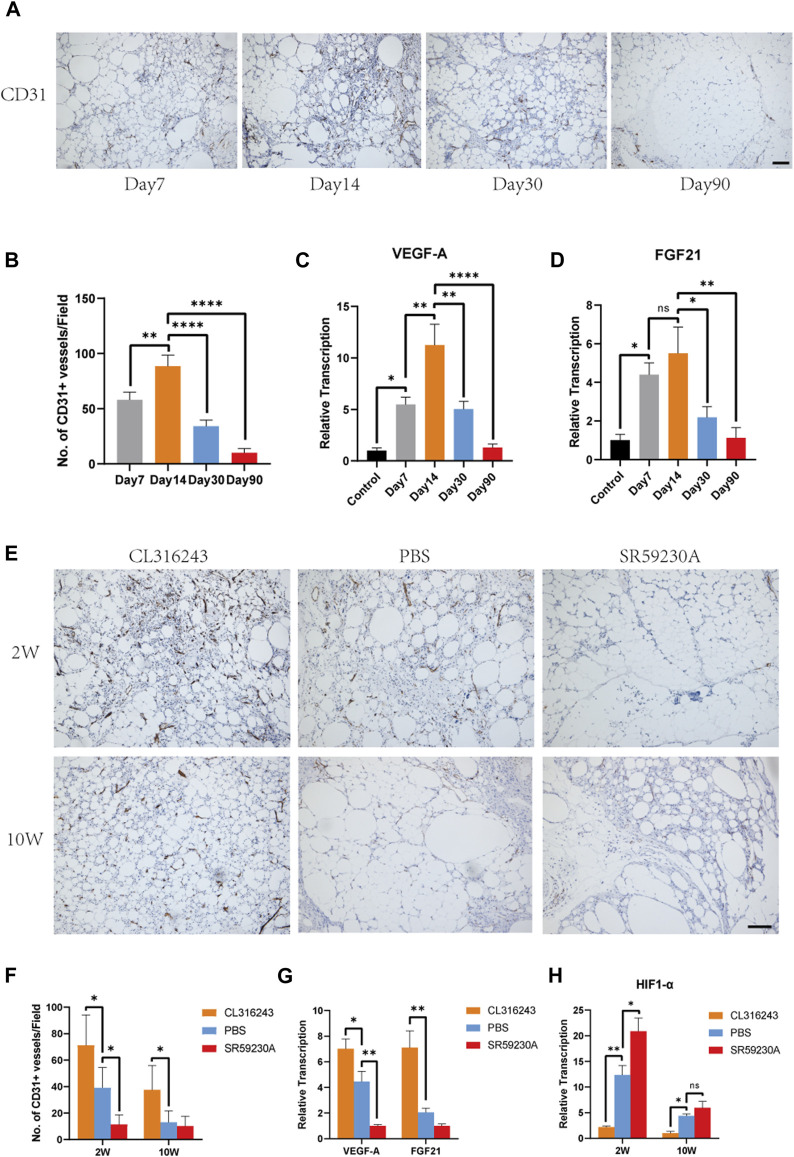
Beige adipocyte formation was associated with early angiogenesis and the production of VEGF-A and FGF21. **(A)** Angiogenesis of fat grafts was superior at day 14 than day 7, 30 and 90. **(B)** Quantification of CD31-positive cells at different time points. **(C** and **D)** Expression levels of angiogenic genes, VEGF-A and FGF21. **(E)** Angiogenesis in the fat grafts 2 weeks and 10 weeks after transplantation, identified using immunohistochemical staining for CD31. **(F)** Number of CD31^+^ vessels at week 2 and week 10. **(G)** Expression of the Vegfa and Fgf21 genes in the fat grafts at week 2, measured using quantitative RT-PCR. **(H)** Expression of HIF1-α associated with hypoxia at week 2 and week 10. (*p < 0.05; **p < 0.01; ****p < 0.0001). Scale bar = 50 μm.

To better determine the relationship between browning and angiogenesis, we evaluated angiogenesis of fat grafts at different time points in the non-treated mice. Immunohistochemistry of CD31 showed that formation of vessels occurred as early as 7 days after grafting and that fat grafts harvested on day 14 contained the most newly formed vessels (Figure 4A). Quantification of newly formed vessels also suggested the superior angiogenesis on day 14 (Day 7, 58.0 ± 3.16 vs. Day 14, 88.4 ± 10.13 vs. Day 30, 34.0 ± 2.569 vs. Day 90, 10.0 ± 1.761, **p < 0.01; ****p < 0.0001) when the browning level was the highest (Figure 4B). There were significantly higher expressions of gene, VEGF-A, on day 14 compared to that at other time points (Figure 4C). However, no significant difference of FGF21 expression was observed between day 7 and day 14 (Figure 4D).

Next, we evaluated the angiogenesis level in the fat grafts treated with CL316243, SR59230A and PBS. Immunostaining using an antibody against CD31 showed that CD31-positive, newly formed vessels were more numerous in the browning upregulation group than in the control group, whereas there was less vascularization in the downregulation group at week 2 (Figure 4E). The mean number of CD31-positive vessels per field in upregulation group was significantly higher than that in the control and downregulation group at postoperative day 14 (Upregulation, 71.2 ± 10.23 vs. Control, 39.2 ± 6.8 vs. Downregulation, 11.4 ± 3.25, *p < 0.05) (Figure 4F). However, ten weeks after transplantation, mean number of CD31-positive vessels was significantly higher than that in the control group but there was no difference between control group and downregulation group (Upregulation, 37.6 ± 8.21 vs. Control, 13.0 ± 3.9 vs. Downregulation, 10.2 ± 3.34, *p < 0.05) (Figure 4F). Furthermore, at week 2, the expression of VEGF-A was significantly higher in the browning upregulation group than in the control group, but significantly lower in the downregulation group (Figure 4G). The expression of FGF21 was higher in the upregulation group than the control group, but there was no significant difference in expression between the control and downregulation groups (Figure 4G). Compared to the control and downregulation group, lower expression of HIF1-α was observed in the upregulation group at week 2 and week 10, which may reflected that superior angiogenesis stimulated by browning agents CL316243 led to reduced hypoxia (Figure 4H).

### 3.5 Browning is Associated with a Reduction in Inflammatory Cell in Grafts

**FIGURE 5:**
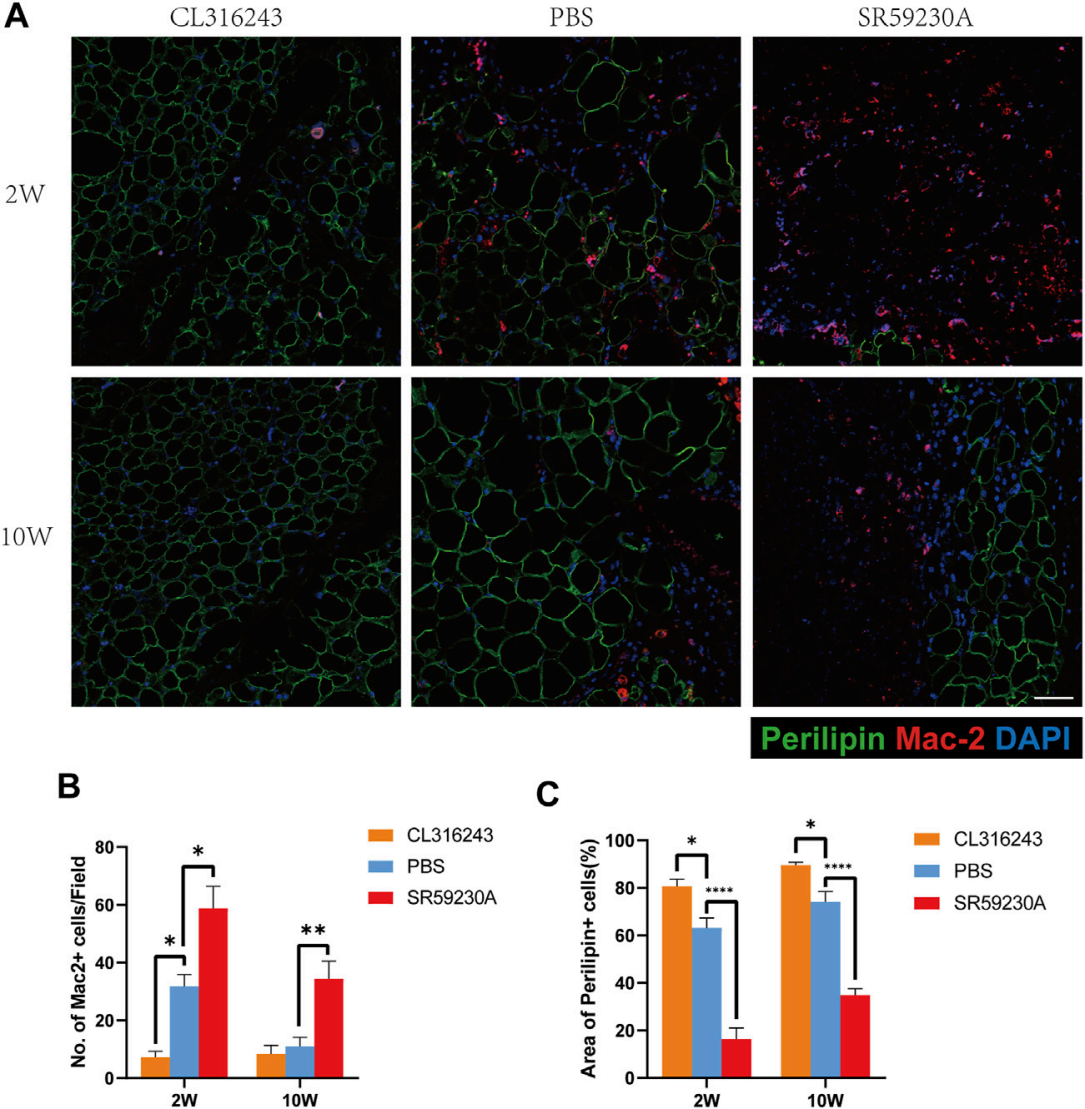
Assessment of adipogenesis and inflammation in fat grafts following transplantation. **(A)** Tissue sections harvested at week 2 and week 10, doubly immunostained for perilipin (green) and Mac2 (red). Fat grafts in the browning upregulation group have the most normal adipose structure, with few macrophages within fat grafts, whereas samples from both the control and browning downregulation groups show severe inflammatory immune cell accumulation at week 2. At week 10, fat grafts in the browning upregulation group and the control group have less inflammatory macrophages in grafts than the downregulation group. Compared with downregulation group, samples in control group and upregulation group have normal adipose structure at week 10. **(B)** Number of Mac2+ cells in each group. **(C)** Area of perilipin positive adipocytes in each group. (*p < 0.05;**p < 0.01;****p < 0.0001) .Scale bar = 50 μm.

2 weeks after transplantation, double-staining of sections with antibodies against perilipin (green) and Mac2 (red) to identify tissue macrophages showed that grafts from the browning upregulation group had a superior structure, and contained more smaller, perilipin-positive adipocytes and fewer Mac2-positive cells than the control group (Figure 5A, above). 10 weeks after transplantation, fat grafts from downregulation group contained more Mac 2-positive cells and less perilipin-positive adipocytes than the upregulation group and the control group. However, no significant difference was observed between the browning upregulation group and the control group. In grafts from the browning downregulation group, there were fewer perilipin-positive adipocytes and a larger number of Mac 2-positive cells, which can be explained by increased tissue macrophage for the purpose of disposing of the dead cells that accumulate because of insufficient angiogenesis (Figure 5A, below). Counting of the Mac2+ cells revealed that they were significantly more numerous in the downregulation group than in the control or upregulation groups at both week 2 (Downregulation, 58.8 ± 7.63 vs. Control, 31.8 ± 4.04 vs. Upregulation, 7.2 ± 2.08, *p < 0.05) and week 10 (Downregulation, 34.4 ± 6.08 vs. Control, 11 ± 3.16 vs. Upregulation, 8.4 ± 2.98, **p < 0.01) but they were significantly less in the browning upregulation groups than in the control group only at week 2 but not week 10 (Figure 5B). Quantification of perilipin-positive adipocytes area showed that 2 weeks and ten weeks after transplantation, perilipin-positive adipocytes in fat grafts from upregulation group occupied larger area per field than the control group while perilipin-positive adipocytes in fat grafts from downregulation group occupied smaller area than the control group (Week 2, Upregulation, 80.7 ± 3.03 vs. Control, 63.09 ± 4.25 vs. Downregulation, 16.33 ± 4.69, *p < 0.05; ****p < 0.0001) (Week 10, Upregulation, 89.52 ± 1.27 vs. Control, 74.09 ± 4.42 vs. Downregulation, 34.75 ± 2.77, *p < 0.05; ****p < 0.0001) (Figure 5C).

## 4 Discussion

Little is known regarding the mechanism underlying fat graft survival/ regeneration after transplantation. Although the important roles of inflammatory cells and ASCs have been recognized, the role of mature adipocytes themselves has been less well researched (Chappell et al., 2015; Cai et al., 2018b; Hong et al., 2018; Liu et al., 2018). Previously, Qiu et al. demonstrated that beige adipocytes are present at the perriphery at 12 weeks after fat grafting (Qiu et al., 2018). Besides, fat grafting induced browning also occurred in patients, as reported by Liu et al. (Liu et al., 2021). Whereas role of this browning observed in human fat grafting is unclear. Therefore, in the present study, for the first time, we aimed to determine the role of browning after grafting and demonstrated that browning in grafts may improve angiogenesis through greater secretion of VEGF-A and FGF21, leading to superior graft retention.

Firstly, we characterized the kinetics of the browning of WAT grafts. However, this finding raised the question of when the browning program of WAT is turned on and how long this lasts. In the present study, we have shown that the beige phenotype of the fat graft changes with time. Beige adipocytes appeared in fat grafts around the regenerating zone as early as 7 days following surgery, peaked in number on day 14, and then became fewer in number until week 12, when they were almost completely absent. This time course of browning suggests that it is a transient state that develops in response to certain stimuli associated with fat grafting, and that after these stimuli are withdrawn, the beige adipocytes lose this phenotype. Similar results were obtained in our previous study, in which the transplantation of tamoxifen-induced beige adipose tissue was followed by whitening of the graft within 12 weeks (Cai t al., 2018a). Indeed, several previous studies have provided evidence that beige or brown adipocytes require sustained stimuli to maintain their BAT phenotype. It has been shown that the transplantation of BAT away from the interscapular region results in a gradual loss of the BAT phenotype (Ferren 1966). Moreover, the browning of adipocytes induced by cold or pharmacologic agents is reversed once these stimuli are withdrawn (Rosenwald et al., 2013). During the early stage of fat engraftment, adipocytes are exposed to hypoxia and inflammation, and metabolites associated with hypoxia and transient inflammatory signaling have been reported to contribute to adipocyte browning (Lee et al., 2013; Carriere et al., 2014; Trayhurn and Alomar 2015; Babaei et al., 2018; Sun et al., 2018). During the later stages of engraftment, the hypoxia and inflammation resolve, which reduces the stimulus for browning. However, the particular browning stimulus in fat grafts has yet to be identified.

Next, we evaluated whether the browning level would affect fat graft retention by pharmaceutical up-/ down- regulation of browning. Experiments by Cai et al., which demonstrated that transplantation of tamoxifen-induced beige adipose tissue improves fat graft retention, along with more recent work that showed that the browning of WAT induced by extracellular vesicles improves fat graft survival, indicate a potential role of beige adipocytes within fat grafts (Cai et al., 2018b; Zhu et al., 2020). However, the physiological significance of the spontaneous browning of fat grafts following transplantation has not been investigated. Here, we have shown that the administration of substances that affect the browning of WAT have effects on the survival and volume of fat grafts. Thus, we have shown for the first time that the survival of fat grafts is affected by the spontaneous browning of WAT *in situ*.

β3-adrenoceptor activation is thought to be a key stimulus for the formation of beige adipocytes (Lee et al., 2012). Therefore, in the present study, we pharmacologically activated or inhibited β3-adrenoceptor activation in a mouse model of fat grafting to further evaluate the role of browning in fat graft regeneration. We found that the administration of the β3 adrenoceptor agonist CL316243 or antagonist SR59230A respectively stimulates or suppresses the browning of fat grafts. The more substantial degree of browning was associated with superior angiogenesis and adipose structure. In the present study, CL316243, which represents a browning stimulator, led to superior angiogenesis and fewer tissue macrophages, and ultimately normal adipose structure with higher fat grafts retention rate.

Based on the fact that beige adipocytes contain multiple, small lipid droplets, and that fat grafts exchange nutrients by means of passive diffusion when avascular, Cai et al. suggested that tamoxifen-induced browning is associated with a higher surface area-to-volume ratio, and thus more efficient diffusion of nutrients and an improvement in survival (Khouri et al., 2014b; Cai et al., 2018a). Thus, it is likely that the browning of white adipocytes is initiated to improve tolerance toward an avascular and hypoxic microenvironment, especially during the early stages of engraftment. It is well known that ASCs and adipocytes can survive in the “survival zone,” the < 300 µm-thick outer part of the graft, whereas both tend to die in the central part, or “necrosis zone”(Eto et al., 2012; Mashiko and Yoshimura 2015). Thus, the activation of browning may be an important means of improving diffusion of nutrients into the graft and improving survival. Surprisingly, as shown above, only a small cluster of adipocytes around the regenerating zone got spontaneous browning after fat grafting, which implies that the browning of the graft might only improve survival in part through more efficient diffusion resulting from a higher surface area-to-volume ratio of the beige adipocytes, and instead may have its effects through additional, as yet unidentified, mechanisms.

In addition to investigating their differing capacity for energy dissipation as heat, investigators have also focused on the contrasting secretomes of white adipocytes and brown/beige adipocytes (Cannon and Nedergaard 2004; Villarroya et al., 2017a; Villarroya et al., 2017b). Evidence obtained in multiple studies suggests that BAT produces VEGF, which improves angiogenesis , and higher vessel density and capillary permeability facilitate triglyceride uptake from the circulation (Fredriksson et al., 2005; Xue et al., 2009; Sun et al., 2014; Mahdaviani et al., 2016). The activation of BAT improves local angiogenesis through greater production of VEGF, which suggests that a factor secreted by brown-like adipocytes in fat grafts may have a positive impact on their survival. Thus, we hypothesized that inducible beige adipocytes in the regenerating zone of fat grafts would have their beneficial effects on survival through a paracrine mechanism. The fact that the beige adipocytes were located in an intermediate zone of the graft might optimize the paracrine/endocrine effects of the beige adipocytes.

When thinking about the factors secreted by beige adipose tissue that might have beneficial effects in fat grafts, we reasoned that VEGF-A and FGF21 would be good candidates because VEGF-A is a “BATokine,” secreted by BAT, and FGF21 can be released by WAT during thermogenic stimulation (Sun et al., 2014; Huang et al., 2017). To determine whether these beige cells act as a source of such factors, as for BAT, we measured the expression of the Vegfa and Fgf21 genes during the early stages of fat engraftment. Interestingly, the data show a positive association between the degree of browning and the production of the pro-vascularization factors VEGF-A and FGF21. VEGF-A is widely accepted as an regulator of physiological angiogenesis (Ferrara et al., 2003). FGF21, a well recognized batokine released by BAT, also exert an positive effect on angiogenesis (Yaqoob et al., 2014; Huang et al., 2019; Zhou et al., 2019; Dai et al., 2021). FGF21 has been reported to normalize glucose and lipid homeostasis, thus preventing the development of metabolic disorders, such as obesity and diabetes (So et al., 2015; So and Leung 2016). Furthermore, FGF21 is also found to exert cell-protective effects in metabolically active organs, such as the liver and pancreas (Wente et al., 2006; Xu et al., 2016). Moreover, increasing studies showed that FGF21 could promote angiogenesis, inhibit oxidative stress and apoptosis in various disease repair model (Yaqoob et al., 2014; Yan et al., 2018; Huang et al., 2019; Zhang et al., 2019; Zhou et al., 2019; Dai et al., 2021). Of note, during fat grafting, great importance should also be attached to functions of FGF21 associated with metabolism. For example, FGF21 increases translocation of glucose transporter 1 (GLUT1) to membrane of adipocytes, which accounts for the increased glucose uptake (Kharitonenkov et al., 2005; Ge et al., 2011). This process might have great influence on survival of adipocytes at early stage after transplantation because of ischemic environment without complete vascular network. Besides, FGF21 could also regulate lipid metabolism and stimulate lipolysis (Inagaki et al., 2007; Hotta et al., 2009). Therefore, FGF21 induced lipolysis could consume the large lipid droplets in adipocytes and thus increased surface area-to-volume ratio, leading superior diffusion to exchange nutrients, as mentioned above. Together, in this context, FGF21 produced by beige adipocytes might serves as not only an angiogenesis stimulator but also an powerful metabolic regulator.

**FIGURE 6:**
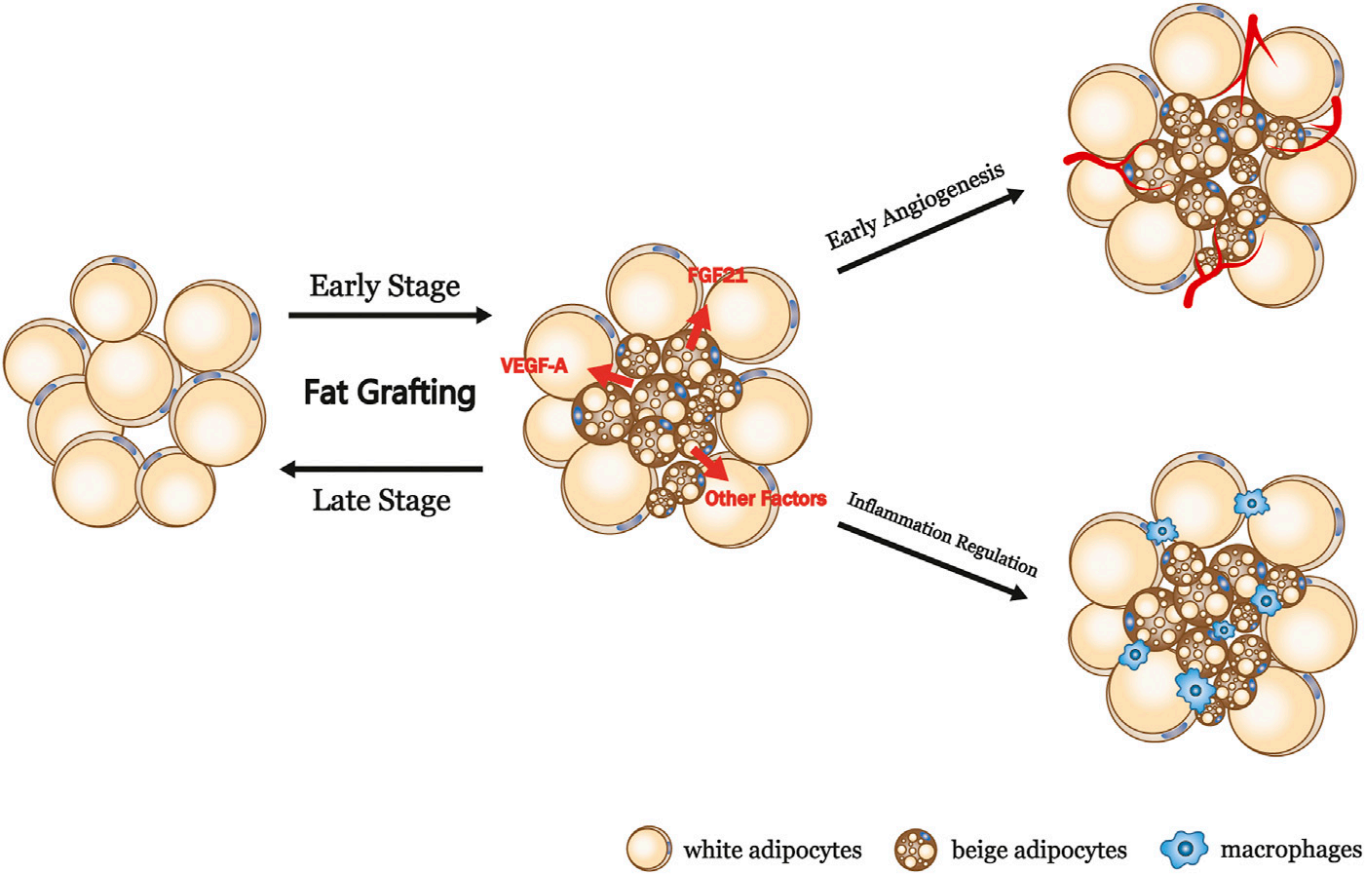
Summary and proposed model of the browning process in fat grafts. VEGF-A, vascular endothelial growth factor A; FGF21, fibroblast growth factor 21.

The results of the present study suggest that beige adipocytes exist in fat grafts and have beneficial effects, particularly in the early stage of engraftment. We speculate that avascularity and hypoxia during the early stage promote the conversion of white to beige adipocytes, which produce more VEGF-A and FGF21, leading to early revascularization (Figure 6). Early revascularization is well known to profoundly affect the long-term outcomes of grafting because a lack of new vessel formation is associated with insufficient nutrient exchange and ultimately cell death (R. J. Khouri et al., 2014a). It is well known that dead cells and cell debris would result in increasing inflammatory macrophages. Conversely, early angiogenesis reduces tissue macrophages which is resulted from improved fat cells survival. The induction of beiging during the early phase of fat engraftment may create a paracrine unit that promotes angiogenesis, leading to a reduction in cell death and relatively moderate inflammation, which contribute to superior graft retention, as shown in Figure 5.

Various browning agents have been used clinically. In the present study, we have highlighted the clinical potential for browning agents, such as CL316243, to improve fat engraftment. Even though the present findings were made in an animal model and the clinical validation of CL316243 requires further studies, mirabegron, a β3-adrenoceptor agonist, has been approved for the treatment of overactive bladder symptoms (Blais et al., 2016; Kelleher et al., 2018). Besides, PPAR gamma agonist rosiglitazone and sildenafil that acts via a nitric oxide-cyclic guanosine monophosphate pathway, which are known as drugs for the treatment of diabetes and erectile dysfunction respectively, were reported to promote expression of genes associated with browning of WAT (Mohanty et al., 2004; Lefterova et al., 2008; Johann et al., 2018; Di Maio et al., 2021). Thus, following further investigations, CL316243 or other promising browning inducers mentioned above might be useful for the stimulation of fat graft browning, with the aim of improving early revascularization and long-term fat graft retention and quality.

Because various signaling pathways, besides β3 adrenoceptor signaling pathways, contribute to browning of WAT under different conditions and the exact mechanism underlying the browning process after fat grafting is unknown, CL316243 and SR59230A applied in the present study may manipulate the degree of browning partly. Since transcriptional regulatory protein PRDM16 appears to control development of beige cells,the use of transgenic mice, such as overexpression or deletion of PRDM16 transgenic mice, would provide better understanding of contribution of browning process to remodeling of fat grafts (Ohno et al., 2012; Cohen et al., 2014). Therefore, mechanism of browning occurred after transplantation and the application of compound that could effectively improve browning clinically might be the focus of our work in the future. Another limitation in this study is that only male animals were used in this study; thus, gender bias should be concerned. Because fat grafting always carried out in women and therefore logically female mice should have priority in this research.

## 5 Conclusion

In the present study, we have shown that the local post-transplantation browning of WAT proceeds along a well-defined time course, and is accompanied by early angiogenesis and superior final graft retention. In a mouse model, treatment with CL316243 for 2 weeks promoted beige adipocyte formation and led to superior fat graft survival. The finding that browning within fat grafts might influence their survival should provoke further research to explore the mechanisms involved in the graft retention and to translate these findings into a clinical means of improving fat graft quality.

## Data Availability

The raw data supporting the conclusions of this article will be made available by the authors, without undue reservation.

## Supplementary Material

Supplementary Figure S1 CL316243 and SR59230A had little effect on body weight and energy expenditure. **(A** and **B)** Both CL316243 and SR59230A did not alter the body weight and food intake of the mice. **(C,D** and **E)** Indirect calorimetry data showing the oxygen consumption, CO_2_ production and heat production of each groups.
