# Corrigendum: Spontaneous browning of white adipose tissue improves angiogenesis and reduces macrophage infiltration after fat grafting in mice

**DOI:** 10.3389/fcell.2022.955768

**Published:** 2022-08-02

**Authors:** Jiayan Lin, Shaowei Zhu, Yunjun Liao, Zhuokai Liang, Yuping Quan, Yufei He, Junrong Cai, Feng Lu

**Affiliations:** Department of Plastic and Cosmetic Surgery, Nanfang Hospital, Southern Medical University, Guangzhou, China

**Keywords:** fat grafting, browning, beige adipocytes, angiogenesis, inflammation

In the original article, there was an error. The symbols “*” and “‡”, indicating correspondence and equal contribution sharing last authorship, were omitted for author Feng Lu.

A correction has been made to the author list:

“Jiayan Lin^†^, Shaowei Zhu^†^, Yunjun Liao^†^, Zhuokai Liang, Yuping Quan, Yufei He, Junrong Cai*^‡^ and Feng Lu*^‡^”

*Correspondence:

Junrong Cai,

drjunrongcai@outlook.com

Feng Lu,

doctorlufeng@hotmail.com

The authors apologize for this error and state that this does not change the scientific conclusions of the article in any way. The original article has been updated.

