# Corrigendum: Spontaneous browning of white adipose tissue improves angiogenesis and reduces macrophage infiltration after fat grafting in mice

**DOI:** 10.3389/fcell.2023.1177893

**Published:** 2023-03-22

**Authors:** Jiayan Lin, Shaowei Zhu, Yunjun Liao, Zhuokai Liang, Yuping Quan, Yufei He, Junrong Cai, Feng Lu

**Affiliations:** Department of Plastic and Cosmetic Surgery, Nanfang Hospital, Southern Medical University, Guangzhou, China

**Keywords:** fat grafting, browning, beige adipocytes, angiogenesis, inflammation

In the published article, there was an error in Figures 4A, E as published. We accidently applied mismatched images to Figures 4A, E. The corrected Figure 4 appears below.

**FIGURE 4:**
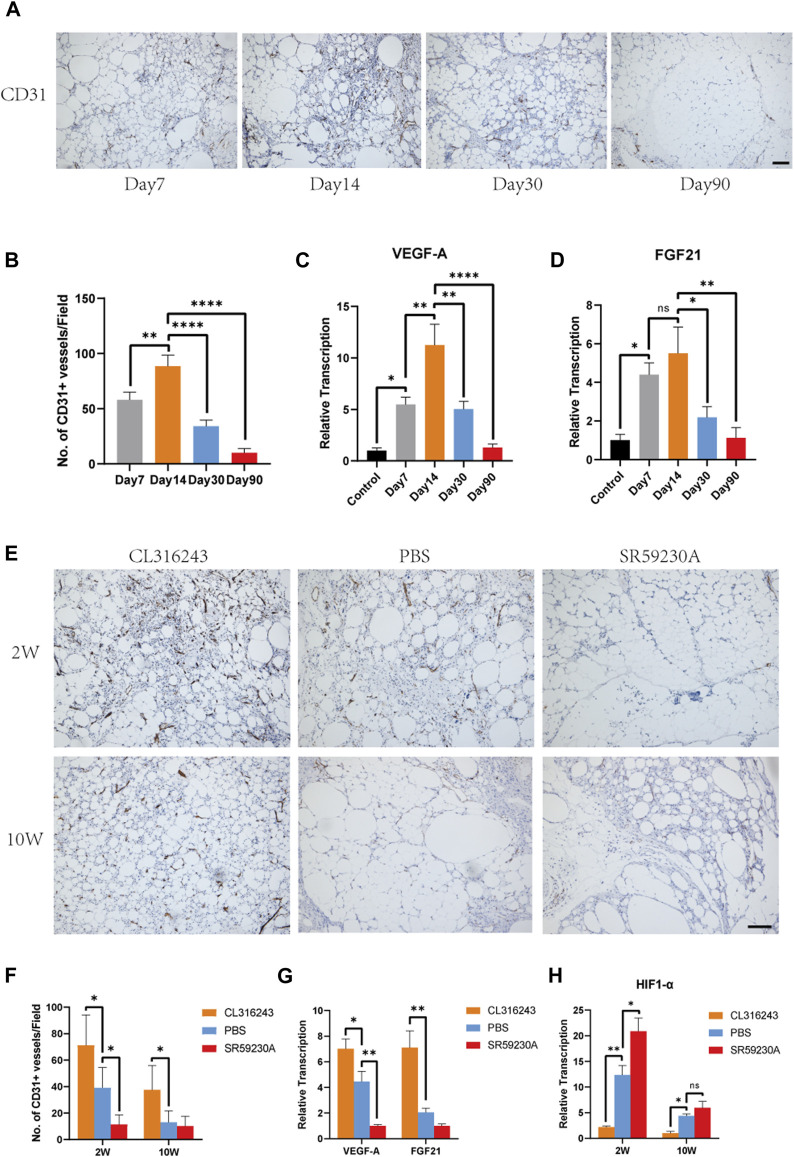
Beige adipocyte formation was associated with early angiogenesis and the production of VEGF-A and FGF21. **(A)** Angiogenesis of fat grafts was superior at day 14 than day 7, 30, and 90. **(B)** Quantification of CD31-positive cells at different time points. **(C, D)** Expression levels of angiogenic genes, VEGF-A and FGF21. **(E)** Angiogenesis in the fat grafts 2 weeks and 10 weeks after transplantation, identified using immunohistochemical staining for CD31. **(F)** Number of CD31+ vessels at week 2 and week 10. **(G)** Expression of the Vegfa and Fgf21 genes in the fat grafts at week 2, measured using quantitative RT-PCR. **(H)** Expression of HIF1-α associated with hypoxia at week 2 and week 10. (*p < 0.05; **p < 0.01; ****p < 0.0001). Scale bar = 50 μm.

The authors apologize for this error and state that this does not change the scientific conclusions of the article in any way. The original article has been updated.

