# Supplementary figures and images for: Spontaneous Browning of White Adipose Tissue Improves Angiogenesis and Reduces Macrophage Infiltration After Fat Grafting in Mice
Source: Front Cell Dev Biol. 2022 Apr 26;10:845158. doi: 10.3389/fcell.2022.845158 (PMC9087586; doi:10.3389/fcell.2022.845158)

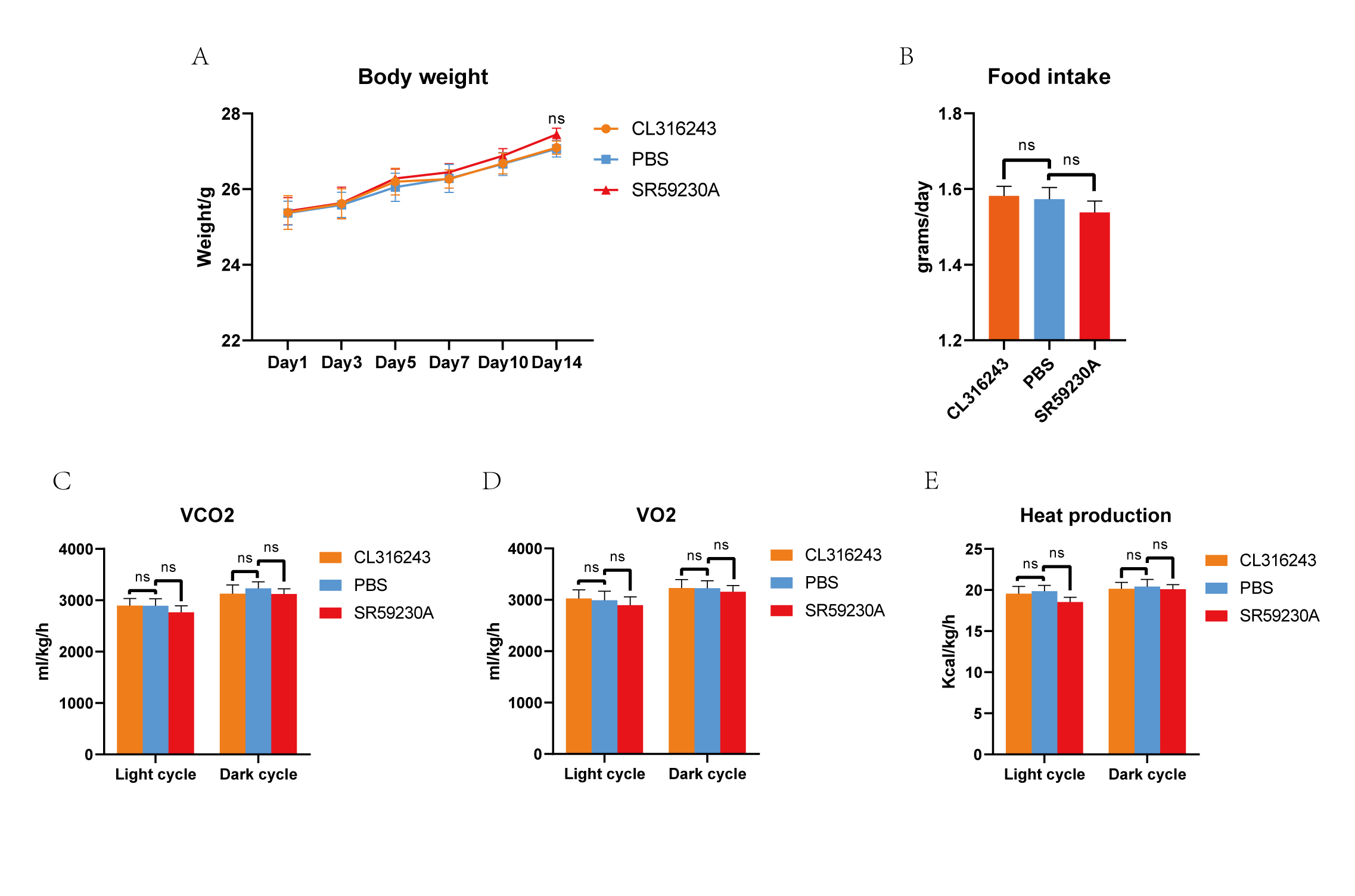

Supplement: Supplementary file 1 [file Image1.tif]
